# A hypoxia‐related lncRNA model for prediction of head and neck squamous cell carcinoma prognosis

**DOI:** 10.1002/cam4.5102

**Published:** 2022-08-03

**Authors:** Junwei Xiang, Yaodong He, Yunshan Li, Kexuan Wu, Mengxiang Cheng, Yuanyin Wang, Ran Chen

**Affiliations:** ^1^ Anhui Medical University, Key Laboratory of Oral Diseases Research of Anhui Province College & Hospital of Stomatology Hefei China

**Keywords:** HNSCC, hypoxia, lncRNA, prognosis, TCGA

## Abstract

**Background:**

Head and neck squamous cell carcinoma (HNSCC) is one of the most common and highly heterogeneous malignancies worldwide. Increasing studies have proven that hypoxia and related long non‐coding RNA (lncRNA) are involved in the occurrence and prognosis of HNSCC. The goal of this work is to construct a risk assessment model using hypoxia‐related lncRNAs (hrlncRNAs) for HNSCC prognosis prediction and personalized treatment.

**Methods:**

Transcriptome expression matrix, clinical follow‐up data, and somatic mutation data of HNSCC patients were obtained from The Cancer Genome Atlas (TCGA). We used co‐expression analysis to identify hrlncRNAs, then screened for differentially expressed lncRNAs (DEhrlncRNAs), and paired these DEhrlncRNAs. The risk model was established through univariate, least absolute shrinkage and selection operator (LASSO), and stepwise multivariate Cox regression. Finally, we assessed the model from multiple perspectives of tumor mutation burden (TMB), tumor immune infiltration, chemotherapeutic sensitivity, immune checkpoint inhibitor (ICI), and functional enrichment.

**Results:**

The risk assessment model included 14 hrlncRNA pairs. The risk score was observed to be a reliable prognostic factor. The high‐risk patients had an unfavorable prognosis and significant differences from the low‐risk group in TMB and tumor immune infiltration. In the high‐risk patients, the common immune checkpoints were down‐regulated, including CTLA4 and PDCD1, and the sensibility to paclitaxel and docetaxel was higher. The functional enrichment analysis suggested that the low‐risk group was accompanied by activated immune function.

**Conclusions:**

The risk assessment model of 14‐hrlncRNA‐pairs demonstrated a promising prognostic prediction for HNSCC patients and can guide personalized clinical treatment.

## Graphical abstract

The risk assessment model of 14‐hrlncRNA‐pairs can use as an effective predictor for prognosis assessment and individualized treatment of HNSCC.

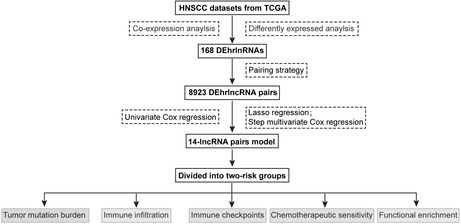

## 1 INTRODUCTION

Head and neck squamous cell carcinoma (HNSCC) mainly refers to tumors of the oral cavity, oropharynx, larynx, nasopharynx, and hypopharynx. More than 930,000 patients with a 50% above mortality rate were diagnosed in 2020.^1^ Despite the continual advancements in surgery, chemotherapy, radiotherapy, and immunotherapy, the 5‐year overall survival rate for HNSCC patients is still poor.^2^ However, even after treatment, HNSCC recurrence and metastasis risk remain high.^3^ Furthermore, the traditional staging approach based on pathological characteristics is incapable of reliably predicting the prognosis of HNSCC patients.^4^ Thus, it is necessary to identify efficient biomarkers for early detection, diagnosis, and prognosis of HNSCC.

Hypoxia‐related pathways were found to be implicated in tumor proliferation, invasion, dissemination, and angiogenesis.^5^ Cancer cells adapt to these processes by regulating the expression of hypoxia‐inducible factors (HIFs).^6^ Hypoxia decreases the effectiveness of chemotherapy by reducing the cellular uptake of drugs and increasing the production of toxic free radicals.^7^ Tumor hypoxia also inhibits immune killing, promotes local immunosuppression, and improves tumor immune tolerance.^8^

Hypoxia‐related lncRNAs play intricate and precise regulatory functions in a variety of tumor biological processes.^9^, ^10^ Under hypoxic conditions, the lncRNA AK058003 enhances gastric cancer spread via targeting gamma‐synuclein.^11^ Another study has demonstrated that lncRNA NORAD could enhance the hypoxia‐induced epithelial‐mesenchymal transition, which promoted pancreatic cancer invasiveness.^12^ HrlncRNAs are also effective in predicting the prognosis of patients with clear cell renal cell carcinoma, bladder cancer, and gastric cancers.^13^, ^14^, ^15^ To the best of our knowledge, the prognostic markers of HNSCC based on hrlncRNA remain un‐explored.

The purpose of this study was to develop a risk prediction model for HNSCC patients utilizing hypoxia‐related lncRNA pairs. First, we screened out hrlncRNAs with significant survival values based on the RNA‐seq data of HNSCC patients. Then, different algorithms, pairing, and iteration were used to construct the model. Finally, we assessed the prediction capacity of the risk score and further explored the differences in TMB, immune microenvironment, and chemotherapeutic sensitivity between the groups. In short, our model can predict HNSCC prognosis effectively and facilitate immunotherapy development and clinical drug selection.

## 2 MATERIALS AND METHODS

### 2.1 Data acquisition and identification of differentially expressed lncRNAs (DEhrlncRNAs)

The RNA‐seq data, corresponding clinical information, and somatic mutation data of HNSCC samples were obtained from TCGA. We selected hypoxia‐related gene sets (M10508, M5466, M2684, M641, M34030) from the Molecular Signatures Database (MSigDB), removed duplicated genes, and included them in subsequent studies analysis. Co‐expression analysis was used to identify hrlncRNAs (|cor| > 0.4, *p* < 0.001).^16^ Afterward, the “limma” package was adopted to identify differently expressed hrlncRNAs between HNSCC and normal tissues with the filter logFC >1.0 and FDR < 0.05.^17^

### 2.2 Establishment of DEhrlncRNA pairs

If lncRNA A had a higher expression level than lncRNA B, we would define “A|B“ as 1; otherwise, the value of “A|B” was 0.^17^ All DEhrlncRNAs were cyclically paired to build a matrix of 1 or 0. A valid match was defined as an lncRNA pair with a value of 0 or 1 accounted for more than 20% of the total number of pairs but less than 80%. The gene pair score was completely calculated based on gene expression profiles within individual tumor samples, which solved the problem of batch correction between samples for us.

### 2.3 Construction of risk assessment model

To obtain all DEhrlncRNA pairs and survival‐related lncRNA pairs (*p* < 0.001), a univariate Cox regression analysis was conducted. To prevent overfitting, we used Least Absolute Shrinkage and Selection Operator (LASSO) regression to obtain the most valuable DEhrlncRNA pairs. Then the risk assessment model was estimated by stepwise multivariate Cox regression. Then, we utilized the “survival ROC” package to produce receiver operating characteristic (ROC) curves to assess the model's prediction capability. Finally, we determined the Akaike information criterion (AIC) values for each point on the 5‐year ROC curve to identify high‐ and low‐risk scores of cut‐off point.

### 2.4 Evaluation of the risk model

Kaplan–Meier survival analysis determined the difference between the two groups in terms of survival to confirm the validity of this cut‐off. To verify the practicability of the generative model for clinical application and to assess the association between risk scores and clinicopathological features, a chi‐square test was performed by the “ComplexHeatmap” package. We also compared the risk scores of various subgroups of clinicopathological characteristics. In order to determine whether the model might serve as an independent clinical prognostic predictor, we utilized univariate and multivariate Cox regression analysis between the risk score and clinicopathological parameters.

### 2.5 Tumor mutation burden (TMB) analysis

We used the “maftools” package to assess the distribution of somatic variants and analyze different gene mutations.^18^ To explore differences in risk scores between mutant and wild‐type groups, we employed the Wilcoxon signed‐rank and plotted box plots.

### 2.6 Immune infiltration analysis

To analyze the association between the risk score and immune infiltration cells, we employed well‐established methodologies to quantify the immune infiltration level of HNSCC patients, including the XCELL, TIMER, QUANTISEQ, MCPCOUNTER, EPIC, CIBERSORT‐ABS, and CIBERSORT. The association between the immune infiltrated immune cell and risk scores was further analyzed by the Spearman correlation analysis. In addition, the single‐sample Gene Set Enrichment Analysis (ssGSEA) was also conducted to examine the variations in immune function between two‐risk groups.

### 2.7 Analysis of immune checkpoints and chemotherapeutic sensitivity

Given the importance of immune checkpoint inhibitors in immunotherapy, we discovered the association between the model and the expression of immune checkpoints with the “limma” and “ggpubr” packages. To assess the clinical application value of the model, we determined the half‐maximal inhibitory concentration (IC50) of primary chemotherapeutic agents by the “pRRophetic” package.^19^ Finally, box plots were used to highlight the variations in chemotherapeutic sensitivity between two‐risk groups.

### 2.8 Function enrichment analysis

We utilized the “clusterProfiler” package to perform Gene Ontology (GO) and Kyoto Encyclopedia of Genes and Genomes (KEGG) enrichment analysis in the patients with low risk, using |NES| > 1.0, *p* < 0.05 as the screening criteria.^20^ The “enrichplot” package visualized the top five functions or pathways of the enrichment.

### 2.9 Statistical analysis

All statistical analyses were performed in R software (Version 4.1.1). Univariate and multivariate analyses were used for Cox regression. Spearman correlation analysis was used to estimate the correlation coefficient of risk score and immune infiltration cell scores. Furthermore, the Wilcoxon test was utilized to analyze the immune checkpoints expression and chemotherapeutic sensitivity between high‐ and low‐risk groups. Unless otherwise stated, *p* < 0.05 was considered statistically significant.

## 3 RESULTS

### 3.1 Identification of DEhrlncRNAs in HNSCC

**FIGURE 1:**
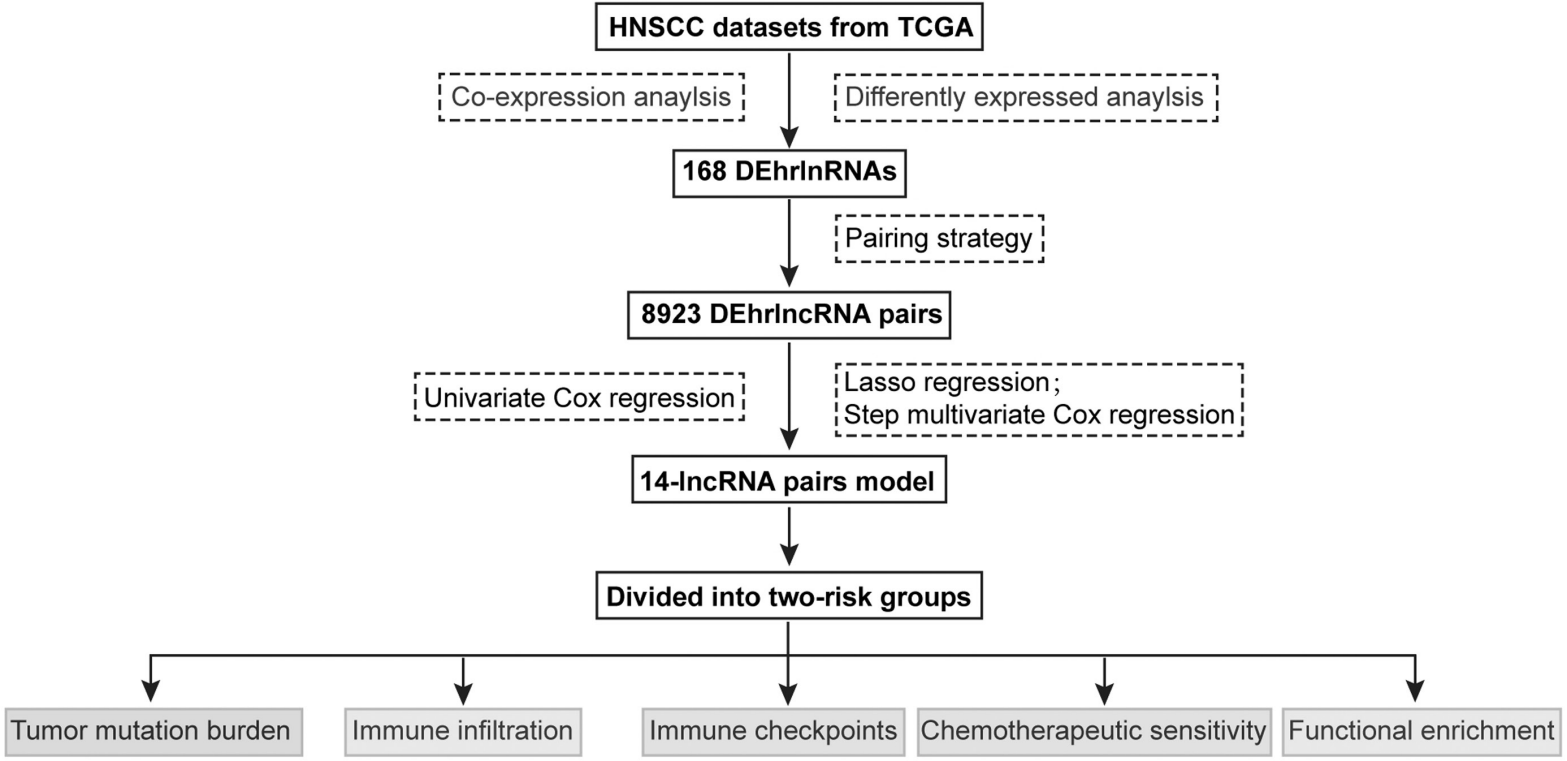
Flowchart of the construction and validation of the hypoxia‐related lncRNA (hrlncRNA) risk assessment model.

**FIGURE 2:**
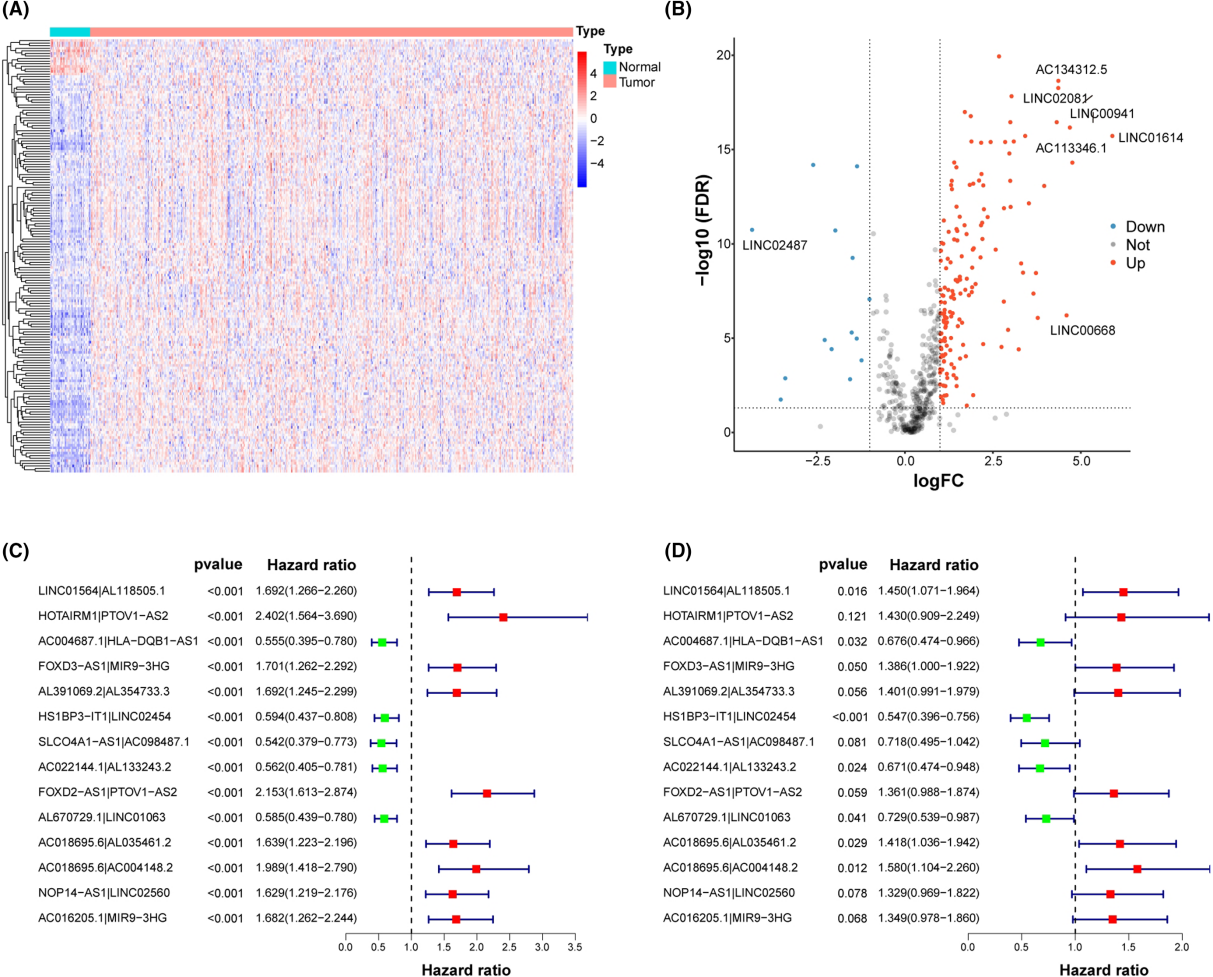
Establishment of the 14‐hrlncRNA‐pair risk assessment model. (A) The heat map of DEhrlncRNAs. (B) The volcano plot of DEhrlncRNAs. (C, D) Forest maps showed that 14 hrlncRNA pairs were subjected to univariate and step multivariate Cox regression.

The flowchart is presented in Figure 1. Firstly, we analyzed the RNA‐seq data of HNSCC patients and extracted a total of 14,142 lncRNAs. And 308 hypoxia‐related genes were identified in the MSigDB, with 305 of them being expressed in HNSCC (Table S1). Among them, HIF1A is a key transcription factor induced by hypoxia, acting as a major regulator of oxygen supply and demand to regulate metabolic reprogramming.^21^ VHL is involved in the regulation of HIF1A protein stability and serves as a repressor of HIF1A transcriptional activity under hypoxic conditions.^22^ In addition, LDHA, TGFA, VEGFA, ANGPT, FGF, and HGF mediate cell proliferation, metabolism, and angiogenesis under the influence of hypoxia.^21^ We then performed a co‐expression analysis and identified 591 hrlncRNAs. As shown in Figure 2A,B, 168 DehrlncRNAs were obtained, including 154 up‐regulated lncRNAs and 14 down‐regulated ones.

### 3.2 Establishment of DEhrlncRNA pairs and a risk assessment model

First, RNA‐seq data and corresponding clinical information of HNSCC were obtained from TCGA, including 39 normal samples and 468 tumor samples. Patients with missing clinical information and an overall survival time of fewer than 30 days were excluded (Table S2). A total of 456 HNSCC patients were included in this study, mainly in advanced stage, including 23 patients with stage I (5.04%), 64 patients with stage II (14.04%), 72 patients with stage III (15.79%), and 240 patients with stage IV (52.63%), and 57 patients with unknown (12.50%). And 168 DEhrlncRNAs in 456 HNSCC patients were subjected to an iterative loop, and 8923 valid DEhrlncRNA pairs were obtained. We determined 109 survival‐related hrlncRNA pairs (*p* < 0.001) (Table S3). The prognostic variables were further screened using LASSO regression analysis, and 32 DEhrlncRNA pairs were obtained (Figure S1A,B). Finally, 14 pairs of hrlnRNA were selected to establish the HNSCC risk assessment model (Figure 2C,D).

**FIGURE 3:**
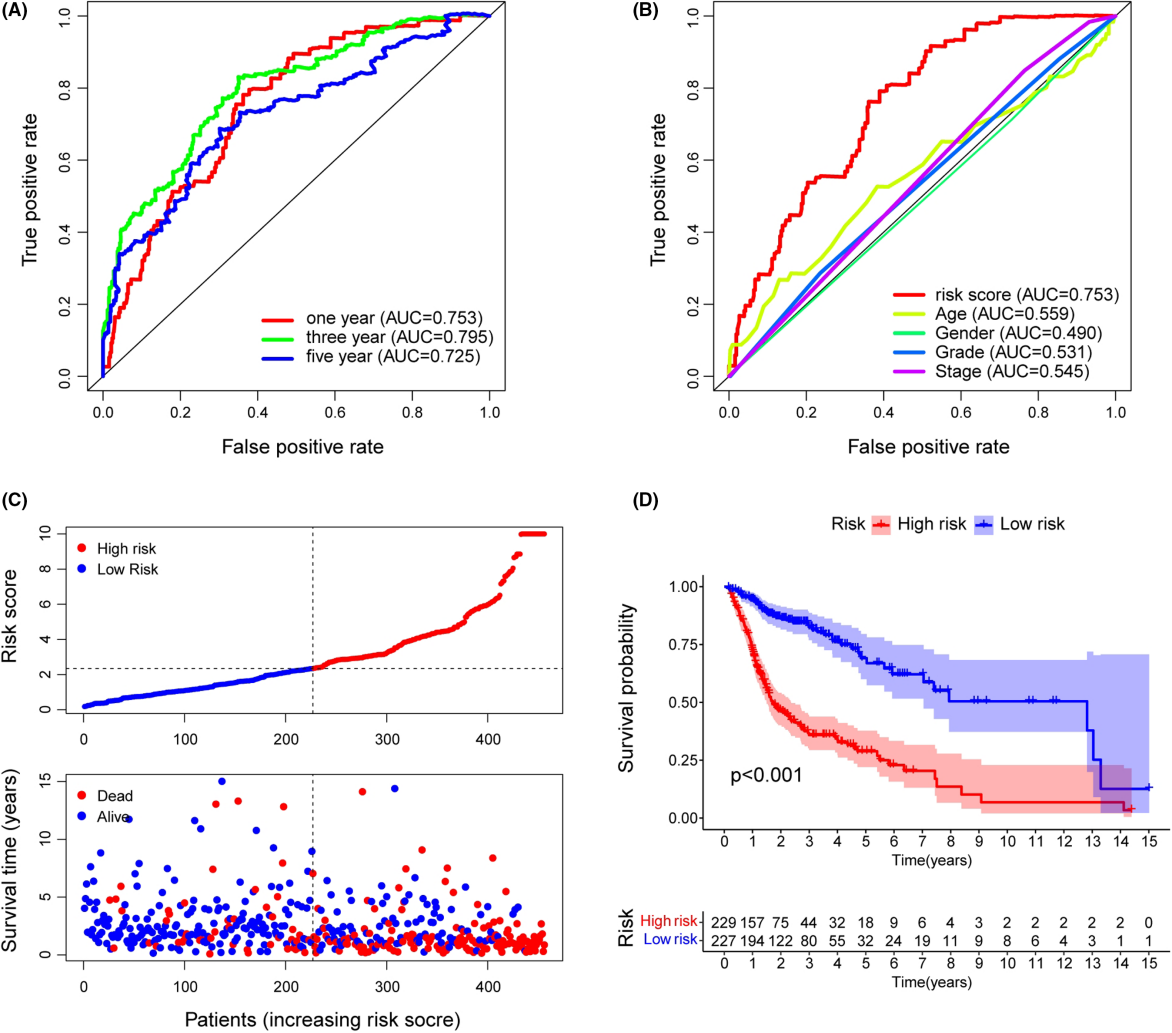
Evaluation of the risk assessment model for prognosis prediction. (A) The AUCs of the 1‐, 3‐, and 5‐year ROC curves. (B) The 1‐year ROC curve of the risk model and other clinicopathological characteristics. (C) The distribution of risk score and survival time and status. (D) The overall survival of the low‐risk groups was higher based on K‐M analysis.

The area under the predicted 1‐year, 3‐year, and 5‐year survival curves were 0.753, 0.795, and 0.725, indicating that the risk assessment model has good sensitivity for survival prediction (Figure 3A). In addition, we selected the maximal inflection point as the cut‐off point and classified patients into low‐ and high‐risk groups (Figure S1C). The risk score outperformed established clinical indicators such as age, gender, tumor grade, and clinical stage in terms of predictive power (Figure 3B).

### 3.3 Clinical evaluation of the risk assessment model

**FIGURE 4:**
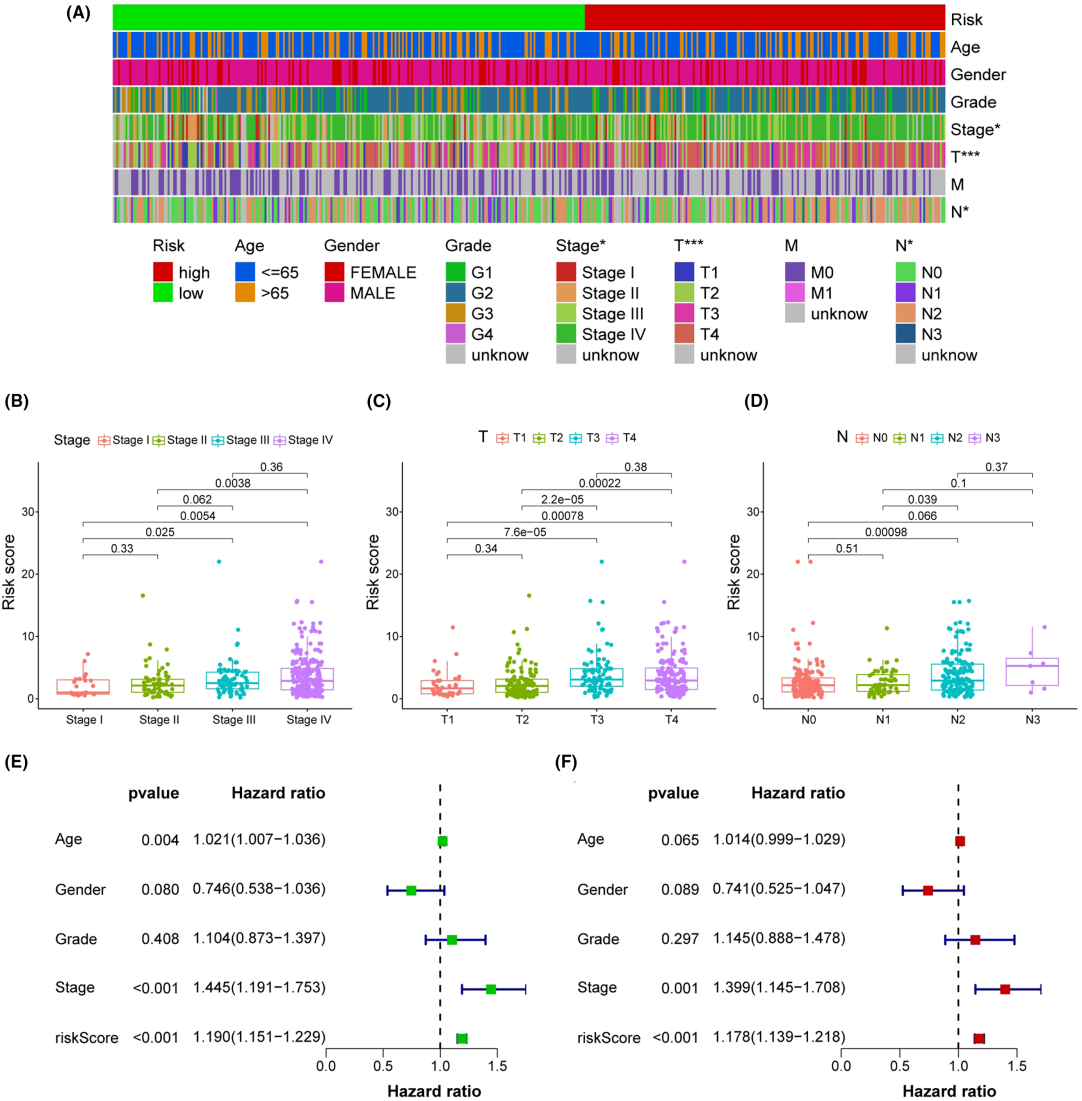
Clinical evaluation of the risk assessment model. (A) The strip chart and the scatter diagram demonstrated that the clinical stage (B), T stage (C) and *N* stage (D) were significantly correlated with the risk score. (E, F) Univariate and multivariate regression analysis showed that the risk score and stage were independent prognostic factors. *p < 0.05, **p < 0.01, and ***p < 0.001.

The 456 samples were separated into two subgroups, with 229 instances in the high‐risk group and the rest in the low‐risk group. The codes and detailed data were presented in Table S4 to reproduce two‐risk groups identification. Figure 3C suggested that the high‐risk group had more death outcomes, indicating that risk scores in HNSCC patients reflected an unfavorable prognosis. As shown in Figure 3D, the patients with higher risk scores experienced a nearly 2‐fold decrease in the overall survival time (*p* < 0.001). Importantly, our model remained a robust prognostic predictor in advanced HNSCC patients (Figure S2). Next, the strip chart and the scatter diagram demonstrated that the risk score was highly related to the clinical stage, T stage, and *N* stage (Figure 4A–D). Age, clinical stage, and risk score were all discovered to be substantial factors for overall survival (Figure 4E). Consistent with previous reports, age was a risk factor for HNSCC prognosis, and gender was not significantly associated with overall patient survival.^23^, ^24^ Finally, the risk score was further verified as an independent and reliable factor in assessing the prognosis of HNSCC patients using multivariate Cox regression (Figure 4F).

### 3.4 Tumor mutational burden analysis

**FIGURE 5:**
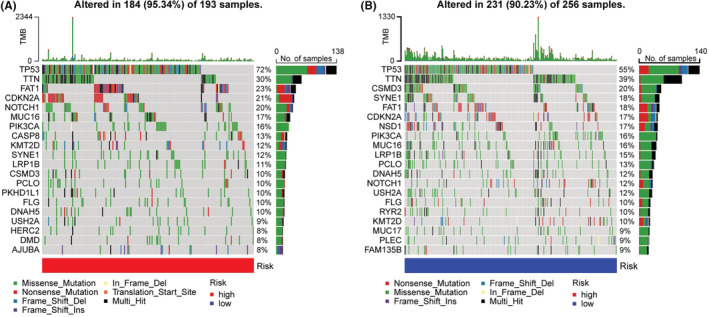
Tumor mutational burden analysis. The waterfall plot of top 20 mutational genes in high‐risk group (A) and low‐risk group (B).

The top 20 genes with the highest frequency of mutations were further investigated (Figure 5A,B). In the high‐risk patients, FAT1, CDKN2A, and NOTCH1 were often mutated genes, whereas, in the low‐risk patients, CSMD3, SYNE1, and FAT1 were frequently mutated genes. We observed that both risk groups had high mutation rates of TP53 and TNN. The risk scores of TP53, NOTCH1, and CASP8 mutations were higher than those of the wild types (Figure S2A), while the risk scores of NSD1, SYNE1, and CSMD3 mutations were lower (Figure S3B). We speculated that different mutations in these genes explained a better prognosis of the low‐risk group.

### 3.5 Relationship between the model and immune cells infiltration

**FIGURE 6:**
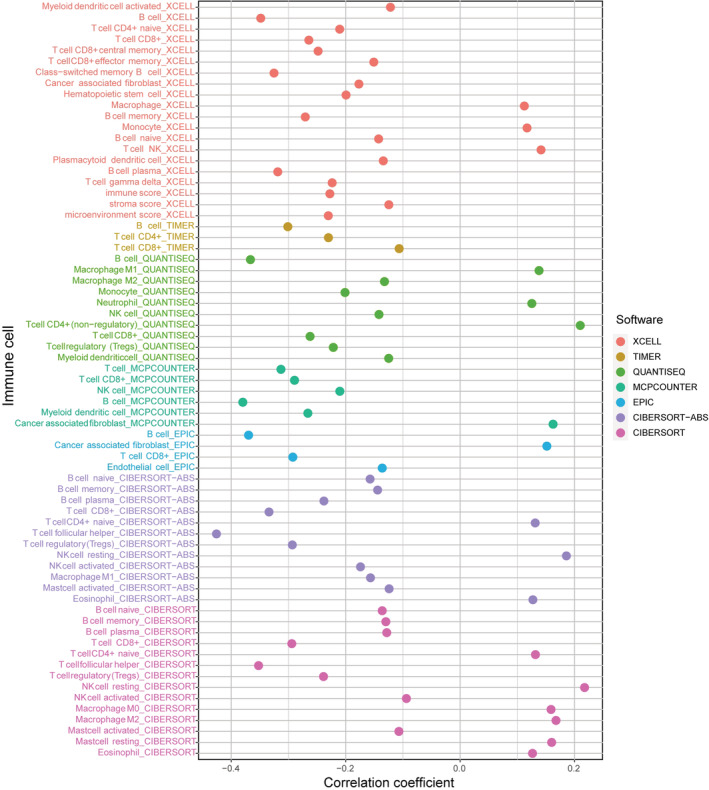
The lollipop chart of immune infiltration correlation showed that the risk score was negatively correlated with T follicular helper cells, B cells, and CD8^+^ T cells.

The association between the risk score and immune infiltration was further analyzed. CD8+ T cells, B cells, and T follicular helper (Tfh) cells were all negatively connected with the risk score, while NK cells, CD4+ T cells, and macrophages were positively correlated (Figure 6). According to the box plot of infiltrating immune cells, CD4, CD8 T cells, resting NK cells, Tfh cells, mast cells, and neutrophils differed significantly between the two risk groups (Figure S4A). Afterward, we used ssGSEA to examine the variations in immune cells, functions, and pathways. The low‐risk patients suggested more active immune‐related activities, particularly B cells, CD8+ T cells, T cell stimulation, Tfh, and TIL (*p* < 0.001) (Figure S4B).

### 3.6 Analysis of ICI‐related molecules, chemotherapeutic sensitivity, and functional enrichment

**FIGURE 7:**
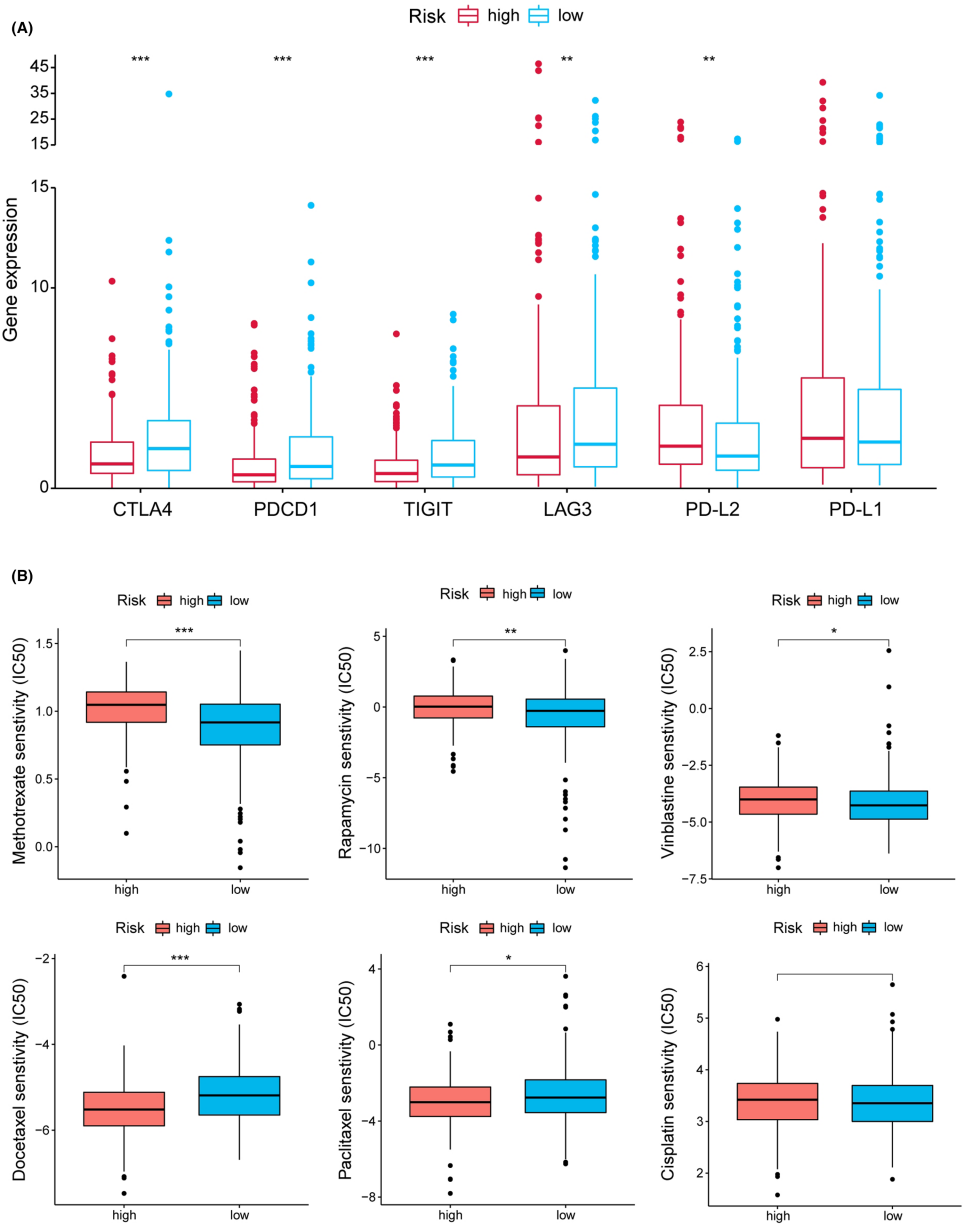
Differences of ICI‐related molecules (A) and chemotherapeutic sensitivity (B) in two‐risk groups. *p < 0.05, **p < 0.01, and ***p < 0.001.

As shown in Figure 7A, CTLA4, PDCD1 (PD1), TIGIT, and LAG3 expression were up‐regulated in the low‐risk group. To explore the potential treatment for HNSCC, the differences in common chemotherapeutic drug sensitivity between two‐risk groups were further evaluated. The high‐risk group revealed a lower IC50 for docetaxel and paclitaxel, while a higher IC50 for methotrexate, rapamycin and vinblastine (Figure 7B). In addition, GO analysis suggested that the low‐risk patients were mainly enriched with immune‐related functions (Figure S5A). Individuals with low risk had significantly enhanced fatty acid metabolism and immune‐related pathways (Figure S5B).

## 4 DISCUSSION

HNSCC is a common squamous cell tumor that can develop anywhere in the head and neck.^25^ Despite advances in cancer treatment, the overall survival rate for HNSCC remains disappointing due to the lack of reliable prognostic biomarkers.^4^ Hypoxia has been confirmed to be closely correlated to the formation of the HNSCC tumor microenvironment, contributing to poor prognosis and chemotherapy resistance.^26^, ^27^ On the other hand, hypoxic modification can significantly improve the HNSCC radiotherapy efficacy.^28^ At present, most research is focusing on developing predictive prognosis models using coding genes or non‐coding RNAs. Due to the differences in detection conditions, platforms, and batches, the expression of each quantified gene has significant heterogeneity. Influenced by the pairing approach of immune‐related lncRNAs,^29^ we attempted to construct a risk assessment model using hypoxia‐related lncRNA pairs. We established an lncRNA pair‐based 0‐or‐1 matrix to assess the risk rather than detecting the transcriptomic expression value, which means better clinical practicability. Herein, the AUC value of the 1‐, 3‐, and 5‐year ROC curves were all over 0.72, indicating that our model was effective in assessing the prognosis. Our model also provided a more comprehensive reference for personalized clinical treatment from immune infiltration, immune checkpoints, and chemotherapeutic sensitivity.

HLA‐DQB1‐AS1 is up‐regulated in HCC tissues, and functional experiments further confirmed that HLA‐DQB1‐AS1, as an oncogenic lncRNA, promotes HCC cell proliferation.^30^ A previous study showed that MIR9‐3HG is a biomarker for cervical cancer development.^31^ In addition, MIR9‐3HG was up‐regulated in HNSCC tissues.^32^ High NOP14‐AS1 expression is associated with poor prognosis in TSCC patients. Functionally, NOP14‐AS1 depletion promotes apoptosis and impedes cell proliferation, migration, and invasion in TSCC.^33^ Zhang et al.^34^ reported that LINC01564 suppresses the inhibitory impact of miR‐107/103a‐3p on phosphoglycerate dehydrogenase gene expression, increasing HCC development. Furthermore, Ke et al. discovered that elevated LINC01564 expression is linked to a worse prognosis in patients with testicular cancer.^35^ FOXD2‐AS1 is elevated in HNSCC tissues and is a potential predictor of HNSCC in patients, promoting the proliferation of HNSCC cell lines.^36^ FOXD3‐AS1 is strongly expressed in a range of disorders, including breast cancer, nasopharyngeal carcinoma, osteosarcoma, melanoma, and thyroid cancer.^37^ Zheng et al.^38^ found that knocking down FOXD3‐AS1 prevented hypoxia‐induced cardiomyocyte damage by up‐regulating the cardioprotective molecule miR‐150‐5p. SLCO4A1‐AS1 is a protective factor for HNSCC prognosis and is significantly associated with overall patient survival.^39^ HOTAIRM1 is down‐regulated in head and neck tumors and neck, as a tumor suppressor gene, inhibits tumor cell proliferation and migration and induces apoptosis.^40^ Hamilton et al.^41^ discovered that HOTAIRM1, which is often downregulated in renal clear cell carcinoma, decreases HIF1 protein levels and attenuates hypoxia‐responsive target genes.

TMB has been demonstrated to be an essential biomarker of response to ICI therapy, and high TMB patients have a higher response to treatment with ICIs.^42^ Our study found significant differences in risk scores between TP53, NOTCH, and NSD1 mutation‐ and wild‐type (*p* < 0.001). TP53 mutations are common in HNSCC patients and closely correlated to poor prognosis.^43^ NOTCH1 is a tumor suppressor gene that regulates the NOTCH pathway and is related to HNSCC occurrence.^44^, ^45^ In line with a recent study, we also identified that HNSCC cell lines with mutant‐type NSD1 have a better chemotherapeutic effect.^46^ Therefore, the impact of TP53, NOTCH1, and NSD1 mutations is consistent with the risk score's prognosis prediction. Moreover, the relationship between these mutations and hypoxia needs further exploration.

Immune regulations are critical in the progression of HNSCC. The subtype and proportion of immune‐infiltrating cells are significant factors affecting immunotherapy response and prognosis. We discovered that Tfh cells, B cells, and CD8+ T cells were strongly negatively related to the risk score, suggesting that these immune cells were relatively abundant in the low‐risk samples. CD8+ T cells are the primary effector cells in anti‐tumor immunity responses, and the level of CD8+ T cell infiltration is correlated with patient favorable survival.^47^ B cells act as both a cancer‐promoter and a tumor‐suppressor in HNSCC.^48^, ^49^ A recent article showed that the high level of Tfh in the tumor microenvironment could reduce patient immunosuppression and improve HNSCC patient survival.^50^, ^51^ Thus, we speculated that these different proportions of immune‐infiltrating cells contributed to the survival differences between the two‐risk groups. The high‐risk groups were more resistant to methotrexate and vinblastine while more sensitive to docetaxel. It provided a new idea for HNSCC chemotherapy. In addition, we discovered that the risk score was inversely associated with the expression level of CTLA4, PDCD1 (PD1), TIGIT, and LAG3, and these checkpoints have been proven to be ICIs treatment markers.^52^, ^53^, ^54^ It suggested that the efficacy of immunotherapy may present an inferior position within the treatment in high‐risk patients.

Compared with other HNSCC models, we have the following advantages. First, our markers are constructed based on the pairing strategy, which can reduce errors due to batch effects and have good clinical application prospects. Second, our model provides a broader exploration of the mechanisms underlying the prognosis differences between high‐ and low‐risk groups. Third, our model is more constructive in the treatment of HNSCC. However, our model also possesses unavoidable disadvantages. First, the approach to marker selection and model construction is not appealing. Besides, the number of our markers is high, which may possess some limitations in practical application. Third, due to objective reasons, our model lacks independent cohort validation, which is where we focus next. Finally, clinical validation of our risk assessment model's clinical application value, such as checkpoint inhibitors and sensitivity to platinum drugs, should also be validated. As the online database did not retrieve the HNSCC cohort containing clinical information and lncRNA sequencing, our next step is to collect enough samples for model validation at multicenter affiliated hospitals.

## 5 CONCLUSION

In conclusion, our hypoxia‐related lncRNA pair model can use as an effective predictor for prognosis assessment and individualized treatment of HNSCC. Differences in tumor mutation burden, immune infiltration microenvironment, and immune checkpoints between the two‐risk groups further elucidated the significance of our predictive model in the development of HNSCC.

## AUTHOR CONTRIBUTION

J.X., Y.H. designed the project and wrote the manuscript. Y.L., K.W. and M.C. performed collection and/or assembly of data, data analysis, and interpretation. Y.W., R.C. gave final approval of manuscript and financial support.

## FUNDING INFORMATION

This work was supported by Anhui Medical University School of Stomatology Discipline Construction Follow‐up Project (2020kqsy02) and Scientific research projects in Anhui universities (YJS20210291).

## CONFLICT OF INTEREST

The authors declare no competing interests.

## ETHICS STATEMENT

This article does not contain any studies with human participants or animals performed by any of the authors.

## Supporting information


Figure S1

Figure S2

Figure S3

Figure S4

Figure S5

Table S1

Table S2

Table S3

Table S4

## Data Availability

The datasets presented in this study can be found in online repositories. The name of the repository can be found below: The Cancer Genome Atlas (TCGA) https://tcga‐data.nci.nih.gov/tcga/.
