# Supplementary figures and images for: A hypoxia‐related lncRNA model for prediction of head and neck squamous cell carcinoma prognosis
Source: Cancer Med. 2022 Aug 3;12(3):3773–85. doi: 10.1002/cam4.5102 (PMC9939198; doi:10.1002/cam4.5102)

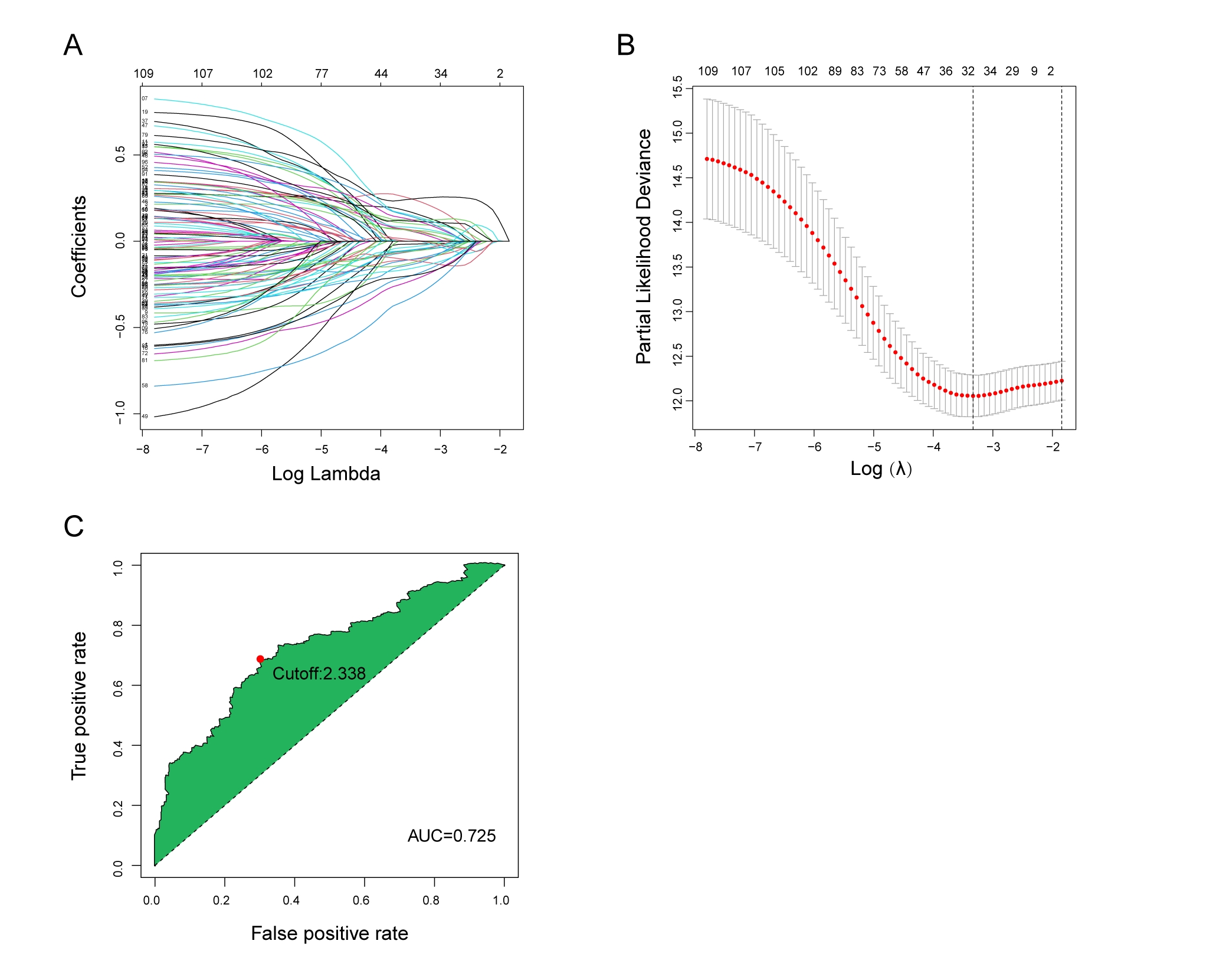

Supplement: Supplementary file 1 — Figure S1 [file CAM4-12-3773-s004.jpeg]

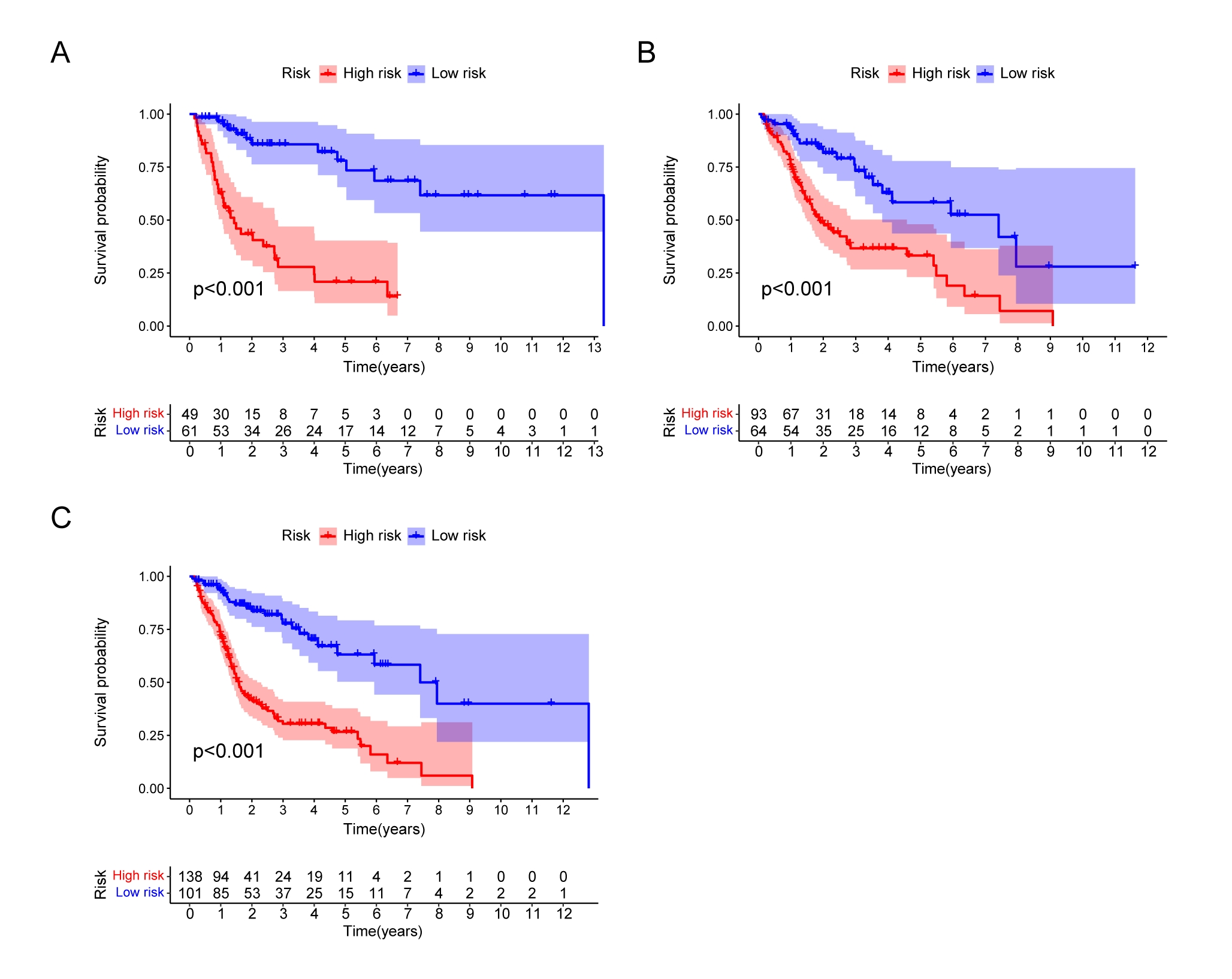

Supplement: Supplementary file 2 — Figure S2 [file CAM4-12-3773-s008.jpeg]

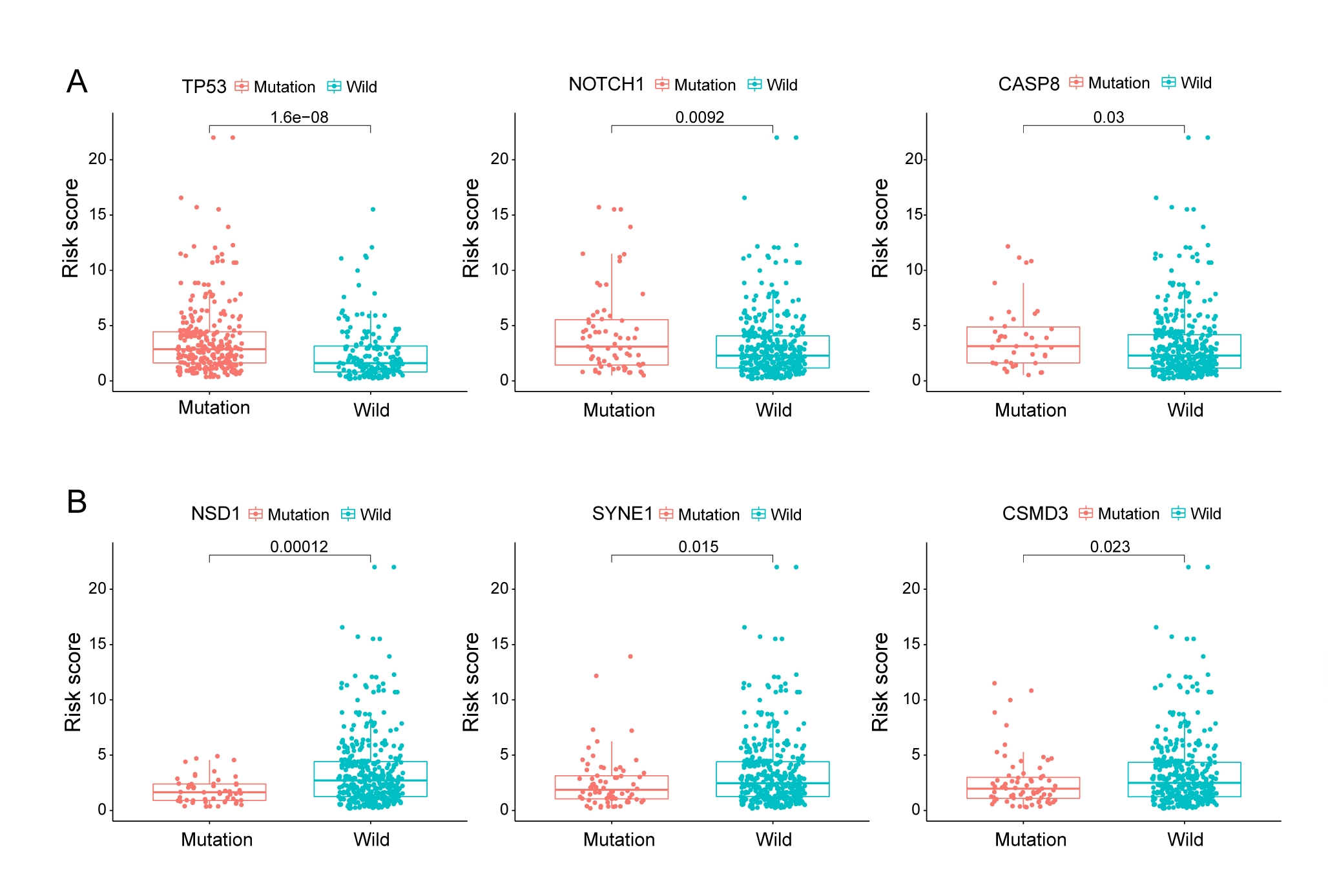

Supplement: Supplementary file 3 — Figure S3 [file CAM4-12-3773-s009.jpeg]

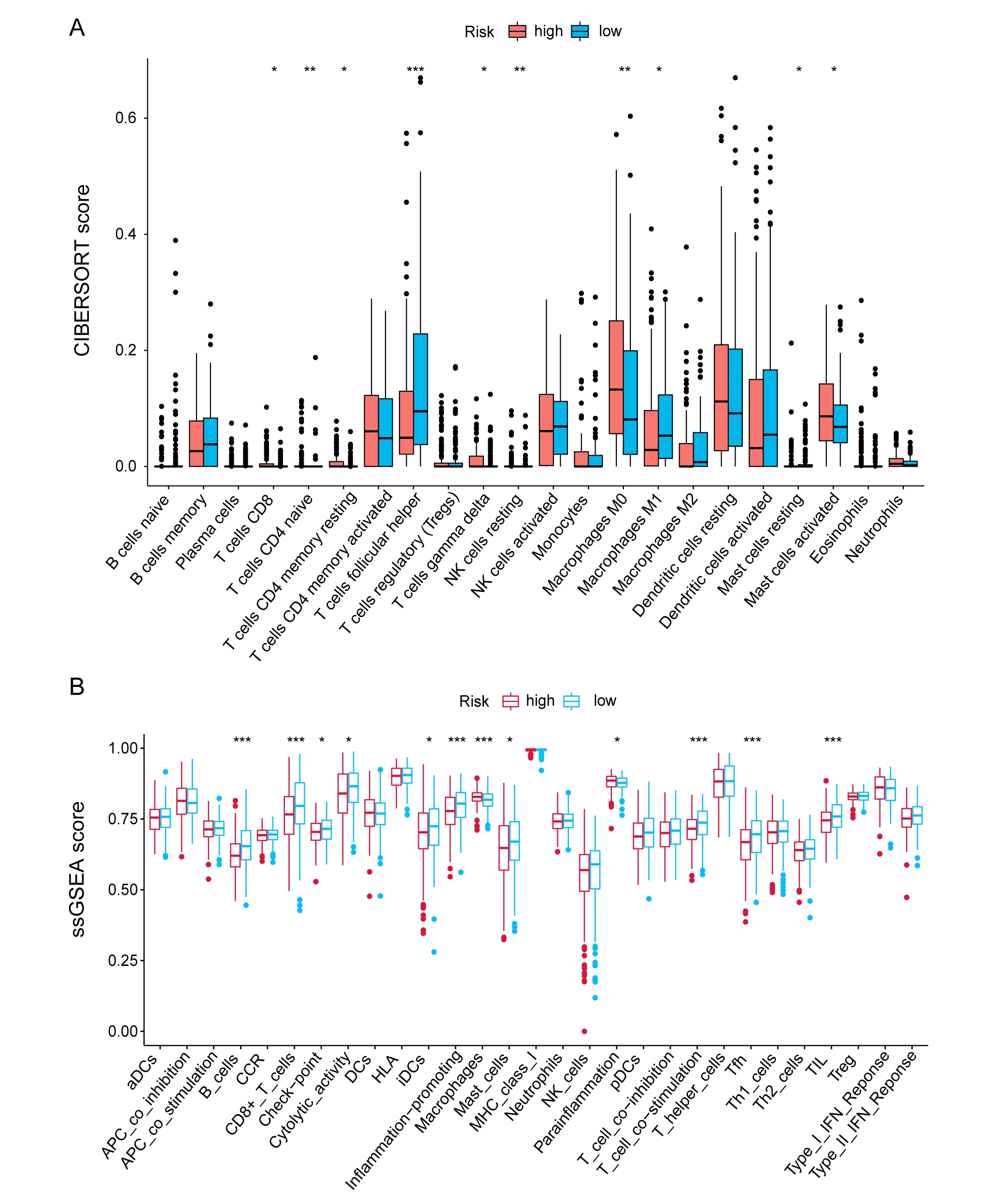

Supplement: Supplementary file 4 — Figure S4 [file CAM4-12-3773-s002.jpeg]

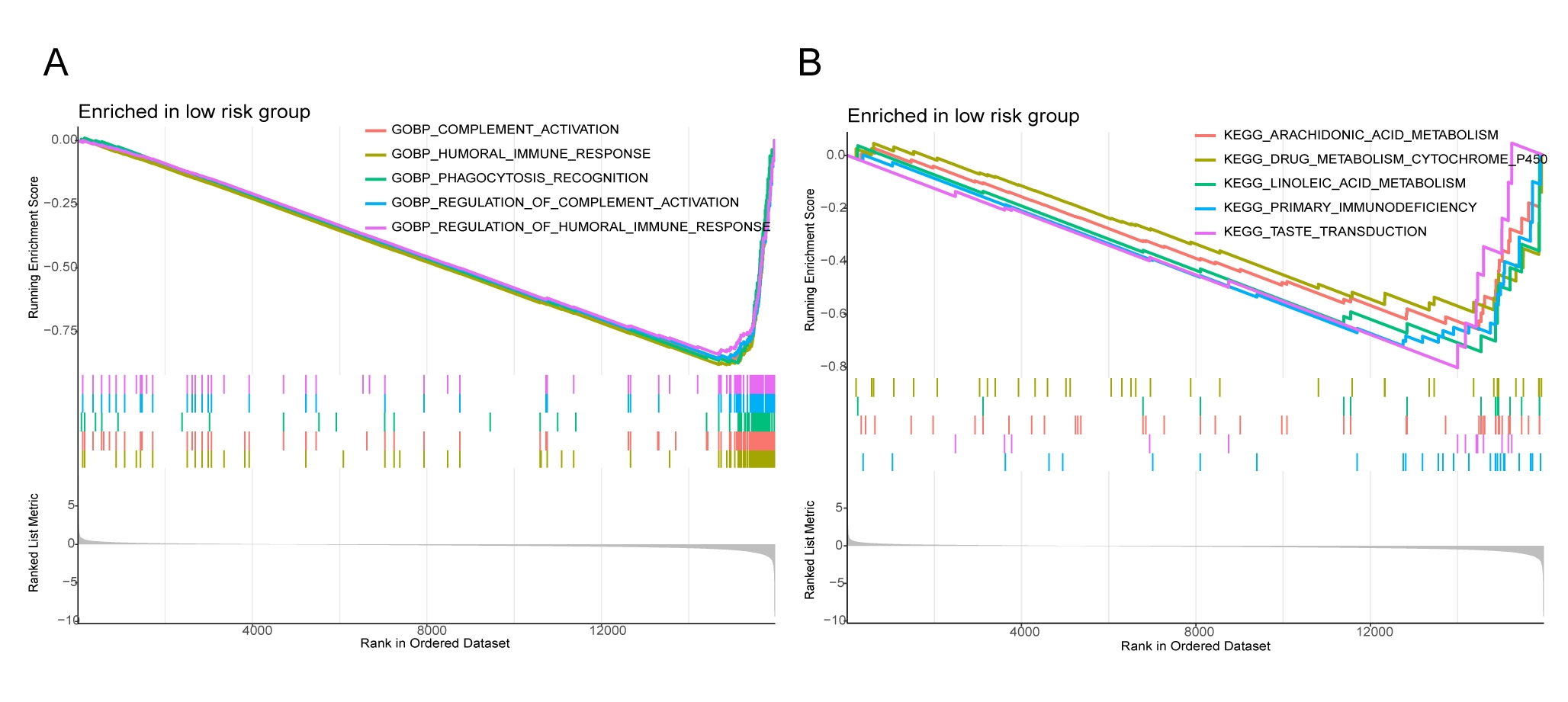

Supplement: Supplementary file 5 — Figure S5 [file CAM4-12-3773-s003.jpeg]
